# Factors Predicting Outcome in Intensive Care Unit-Admitted COVID-19 Patients: Using Clinical, Laboratory, and Radiologic Characteristics

**DOI:** 10.1155/2021/9941570

**Published:** 2021-07-07

**Authors:** Aminreza Abkhoo, Elaheh Shaker, Mohammad-Mehdi Mehrabinejad, Javid Azadbakht, Nahid Sadighi, Faeze Salahshour

**Affiliations:** ^1^Department of Radiology, School of Medicine, Advanced Diagnostic and Interventional Radiology Research Center (ADIR), Imam Khomeini Hospital, Tehran University of Medical Sciences, Tehran, Iran; ^2^Non-Communicable Diseases Research Center, Endocrinology and Metabolism Population Sciences Institute, Tehran University of Medical Sciences, Tehran, Iran; ^3^Department of Radiology, Faculty of Medicine, Kashan University of Medical Sciences, Kashan, Iran

## Abstract

**Purpose:**

To investigate the factors contributing to mortality in coronavirus disease 2019 (COVID-19) patients admitted in the intensive care unit (ICU) and design a model to predict the mortality rate.

**Method:**

We retrospectively evaluated the medical records and CT images of the ICU-admitted COVID-19 patients who had an on-admission chest CT scan. We analyzed the patients' demographic, clinical, laboratory, and radiologic findings and compared them between survivors and nonsurvivors.

**Results:**

Among the 121 enrolled patients (mean age, 62.2 ± 14.0 years; male, 82 (67.8%)), 41 (33.9%) survived, and the rest succumbed to death. The most frequent radiologic findings were ground-glass opacity (GGO) (71.9%) with peripheral (38.8%) and bilateral (98.3%) involvement, with lower lobes (94.2%) predominancy. The most common additional findings were cardiomegaly (63.6%), parenchymal band (47.9%), and crazy-paving pattern (44.4%). Univariable analysis of radiologic findings showed that cardiomegaly (*p* : 0.04), pleural effusion (*p* : 0.02), and pericardial effusion (*p* : 0.03) were significantly more prevalent in nonsurvivors. However, the extension of pulmonary involvement was not significantly different between the two subgroups (11.4 ± 4.1 in survivors vs. 11.9 ± 5.1 in nonsurvivors, *p* : 0.59). Among nonradiologic factors, advanced age (*p* : 0.002), lower O_2_ saturation (*p* : 0.01), diastolic blood pressure (*p* : 0.02), and hypertension (*p* : 0.03) were more commonly found in nonsurvivors. There was no significant difference between survivors and nonsurvivors in terms of laboratory findings. Three following factors remained significant in the backward logistic regression model: O_2_ saturation (OR: 0.91 (95% CI: 0.84–0.97), *p* : 0.006), pericardial effusion (6.56 (0.17–59.3), *p* : 0.09), and hypertension (4.11 (1.39–12.2), *p* : 0.01). This model had 78.7% sensitivity, 61.1% specificity, 90.0% positive predictive value, and 75.5% accuracy in predicting in-ICU mortality.

**Conclusion:**

A combination of underlying diseases, vital signs, and radiologic factors might have prognostic value for mortality rate prediction in ICU-admitted COVID-19 patients.

## 1. Introduction

Few months after the first reports of coronavirus disease 2019 (COVID-19), it was declared a pandemic [1]. Given its high transmissibility, SARS-CoV-2 has infected millions of people worldwide and has placed a huge burden on the healthcare system [2]. Some infected patients develop acute respiratory distress syndrome (ARDS), multiple organ failure, pulmonary embolism, and heart failure [3–5]. ARDS is the most common reason for intensive care unit (ICU) admission in these patients [6, 7]. For patients requiring intensive care, ICU admission occurs about 10 days after the onset of symptoms and 14 days after infection [8]. After a rapid surge in COVID-19 cases, the need for intensive care and aggressive treatment has been dramatically increased around the world [9]. The in-ICU mortality rate of COVID-19 is twice that of other causes of viral pneumonia that require ICU admission [10].

Although the gold standard test to diagnose COVID-19 is real-time reverse-transcription polymerase chain reaction (rRT-PCR), the rate of false-negative results is high, especially in the early stages of the disease. Some studies showed a median false-negative rate of 38% for the rRT-PCR test on the first day postsymptom onset [11, 12]. Chest CT scan is not only a diagnostic modality with high sensitivity (92%), especially in uncertain cases, but is also of prognostic value [13, 14]. Some reports claimed that the accuracy of CT scan is higher than that of rRT-PCR in detecting COVID-19 [13, 15]. Several studies showed that factors like advanced age, obesity, and comorbidities such as hypertension (HTN) and diabetes mellitus are associated with higher mortality in COVID-19 cases [16–18]. About one-third of the hospitalized COVID-19 patients will eventually need ICU admission [19, 20].

As the knowledge on predictors of worse outcomes in COVID-19 ICU patients is limited, we aimed to conduct this clinical study in an attempt to find and describe risk factors related to the mortality of critically ill ICU-admitted COVID-19 patients.

## 2. Methods and Materials

### 2.1. Study Design and Participants

The present study was reviewed and approved by the Institutional Review Board of our institute. The written informed consent was waived regarding the retrospective design of the study (IR.TUMS.IKHC.REC.1399.054). The participants' medical records were retrieved from the institution's registry of COVID-19 patients.

We included patients admitted to ICU with rRT-PCR (performed on specimens collected from nasopharyngeal or oropharyngeal secretions) confirmed COVID-19 infection and a definite outcome (death or discharge) from September to October 2020. All patients underwent an on-admission chest CT scan and had the required medical documents for this study already registered. Participants were divided into two subgroups: survivors and nonsurvivors. The demographic, clinical, laboratory, and radiologic characteristics of these two groups were enlisted and compared. All ICU admission criteria and treatment regimens were based on the latest version of the related national protocols.

### 2.2. Data Acquisition

Data collectors retrieved “patients” information from electronic and paper records. Data collection included (a) demographic information: age and sex; (b) vital signs: temperature (T, Celsius), oxygen saturation (SpO_2_), heart rate (HR) per minute, respiratory rate (RR) per minute, and blood pressure (BP, mmHg); (c) comorbidities: hypertension (HTN), diabetes (DM), chronic obstructive pulmonary disease (COPD), immunocompromised conditions (hereditary or acquired immunodeficiency diseases, chemoradiation therapy, and long-term corticosteroid usage), and hypothyroidism; (d) laboratory test results: white blood cell counts including neutrophil and lymphocyte counts, hemoglobin, platelet, creatinine, urea, international normalized ratio (INR), partial thromboplastin time (PTT), D-dimer, lactate dehydrogenase (LDH), C-reactive protein (CRP), and pro-B-type natriuretic peptide (Pro-BNP); and (e) radiologic findings (discussed further in the following sections). All vital signs and laboratory results were gathered on admission. In addition, hospital length of stay (separately for in-ward and in-ICU stay) has been evaluated.

### 2.3. Image Acquisition and Interpretation

All CT examinations were performed using either 6 or 16 slices (Siemens SOMATOM Emotion, Erlangen, Germany) MDCT scanner. Imaging parameters were set as follows: tube voltage of 130 kVp, tube current of 70 mAs, slice width of 2–5 mm, beam collimation of 1.2 mm, and tube rotation time of 0.6 seconds, reconstructed with a mediastinum B20f smooth kernel and a lung B70f sharp kernel (Siemens Healthineers, Erlangen, Germany) with a reconstructed slice thickness of 1.2 mm; coronal and sagittal multiplanar reconstructions were also available. All CT images were obtained without contrast injection at the time of presentation, in the supine position, and full inspiration as tolerated by the patients.

Two board-certified diagnostic radiologists, with 9 and 13 years of experience in thoracic radiology and blinded to patients' clinical data, independently interpreted chest CT scans, in both lung and mediastinal windows. Intraclass correlation coefficient (ICC) was calculated to assess interrater reliability. If ICC < 0.8, in case of any disagreement in image interpretation, the discrepancy was resolved by consensus. If ICC ≥ 0.8, the values reported by the radiologist with higher experience were recorded. Chest CT scan features were reported and described based on the Fleischner Society glossary and published literature on viral pneumonia [21, 22]. CT features include the following: (a) predominant pattern: ground-glass opacity (GGO) and consolidation; (b) dominant distribution: peripheral, axial, and diffuse; (c) the number of involved lobes; (d) laterality: unilateral or bilateral involvement; (e) lower lobes involvement; (f) additional findings: cardiomegaly, pleural effusion, pericardial effusion, dilated pulmonary trunk, and pleural thickening; and (g) other morphologies: parenchymal band, crazy paving, and reverse halo.

A semiquantitative scoring system was exploited to evaluate the pulmonary involvement (PI) status. All five lung lobes were reviewed for GGO and consolidation. Each lobe was scored between 0 and 5 based on involvement percentage (0: no involvement; 1: <5%; 2: 6–25%; 3: 26–50%, 4: 51–75%; and 5: >76%). Each lobe could score 5 points at maximum; thus, the total score ranges from 0 to 25. Accordingly, the PI density index equals the total PI score divided by the number of involved lobes.

### 2.4. Statistical Analysis

Categorical variables were reported with their counts and percentage, and continuous variables were presented as means (with standard deviation (SD)). All statistical analyses were performed in the SPSS for Windows (version 16, Chicago, IL, USA). The normality of the data was evaluated by the Kolmogorov–Smirnov test. Univariable analyses (either *t*-test, Mann–Whitney *U* test, or cross-tabulation) were used in the first place for the primary variables. All variables with *P* < 0.1 were then entered into a multiple logistic regression model with a backward approach to adjust for collinearity and covariance. Sensitivity, specificity, positive predictive value (PPV), negative predictive value (NPV), and accuracy (and their 95% confidence intervals (CIs)) were calculated for combinations of 3 significant findings. *P* < 0.05 was considered significant.

## 3. Result

### 3.1. Patients' Characteristics and Clinical Findings

In this study, 121 ICU-admitted rRT-PCR-confirmed COVID-19 patients with a mean age of 62.2 ± 14.0 years (range, 25–90) were included; of them, 82 (67.8%) were male. 41 patients (33.9%) survived, and the rest succumbed to death. Of all participants, 74 (61.1%) ICU patients were intubated and 60 (81%) of them could not survive. Noteworthy, survivors were 8.3 years younger than nonsurvivors (56.7 ± 11.7 vs. 65.0 ± 14.33, *p* : 0.002). There was no significant difference between the survival rates of ICU-admitted males and females. However, men were twice as likely to be admitted to the ICU (67.8% vs. 32.2%).  Table 1 summarizes the demographic and clinical characteristics of survivors and nonsurvivors.

Hypertension (41.3%) and diabetes mellitus (35.6%) were the most common comorbidities found in ICU patients; however, only the rate of HTN was significantly higher in nonsurvivors compared to survivors (35 ± 47.9 vs. 8 ± 25.8%, *p* : 0.03). Regarding vital signs, SpO_2_ (85.8 ± 5.3 vs. 81.2 ± 10.2, *p* : 0.01) and diastolic blood pressure (78.2 ± 10.9 vs. 72.0 ± 13.0, *P* : 0.02) were significantly lower in the deceased group. Survivors and nonsurvivors did not differ significantly by the hospital length of stay (15.8 ± 6.8 days for survivors vs. 15.2 ± 10.0 days for nonsurvivors, *p* : 0.80). Patients who did not survive from COVID-19 stayed 4.3 days longer in the ICU, but it was not statistically significant (6.0 ± 2.1 vs. 10.3 ± 10.0, *p* : 0.10). Comparing laboratory findings, there was no difference between the survivor and nonsurvivor groups (Table 1).

### 3.2. Radiologic Findings

The most common radiologic patterns observed were GGO (71.9%) with peripheral (38.8%) and bilateral (98.3%) involvement, with lower lobes (94.2%) predominancy. The most common additional findings were cardiomegaly (63.6%), parenchymal band (47.9%), and crazy-paving pattern (44.4%). The mean total PI score (11.4 ± 4.1 vs. 11.9 ± 5.1, *p* : 0.59) and PI density index (2.4 ± 0.7 vs. 2.4 ± 0.9, *p* : 0.90) were not meaningfully different between survivors and nonsurvivors. The radiologic findings that showed a significant difference in frequency between the two subgroups were cardiomegaly (51.2% of survivors vs. 70.0% of nonsurvivors, *p* : 0.04), pleural effusion (12.2% vs. 31.3%, *p* : 0.02), and pericardial effusion (2.4% vs. 15.0, *p* : 0.03). However, the distribution pattern was not significantly associated with mortality (*p* : 0.59) (Table 2).

After incorporating the significant variables into the backward logistic regression model, three of them remained significant: higher SpO_2_ as a protective factor and pericardial effusion and HTN as predisposing factors for death (Table 3). The regression model was statistically significant (*χ*^2^ (3) = 19.9, *p* < 0.001). The model explained 26.2% (Nagelkerke *R*^2^) of the variance in death. Hosmer–Lemeshow test showed that this model was fitted well to the data (*χ*^2^ (8) = 5.6, *p* : 0.69). This model had a 78.7% (68.2%–87.1%) sensitivity, 61.1% (35.7%–82.7%) specificity, 90.0% (83.3%–94.2%) PPV, 39.3% (27.0%–53.1%) NPV, and 75% (66.7%–83.6%) accuracy.

## 4. Discussion

The main finding of this study is that the best approach for mortality prediction in COVID-19 ICU patients is a combination of the underlying diseases, vital signs, and radiologic features. Among the radiologic findings studied, pericardial effusion was associated with mortality. Moreover, oxygen saturation and hypertension were the prognostic factors among other clinical factors that reached the statistical significance threshold. Other factors and their effects are believed to be minimal. The model can help physicians detect high-risk patients earlier to set up their therapeutic/follow-up schedule beforehand.

Male gender was associated with higher hospitalization, ICU admission, and need for mechanical ventilation [23, 24]. Yet, the ICU mortality rate was not gender-dependent. The overall mortality rate in studies on ICU patients has been reported to be somewhere between 16% and 78% [25]. This wide gap in reported mortality rate can be due to the difference in the severity of disease at ICU admission time, availability of ICU beds, ICU admission criteria, sample size, underlying conditions, and length of follow-up. Half of our study sample had HTN and or DM that shows their important role in ICU admission. Like the current study, HTN was the most common comorbidity in COVID-19 patients in other research studies [25–27]. Although with aging, the mortality rate increases, part of this notion seems to come indirectly from the commonness of underlying medical conditions in older adults [19].

In a systematic review, typical chest CT findings of critically ill COVID-19 patients were GGO, consolidative opacities, multilobar, and bilateral pulmonary involvement, consistent with our findings [28]. Unilateral and unifocal involvements were more commonly found in the early stages of the disease and thus are not usually encountered in chest imaging of ICU-admitted patients [29]. Expansion of the GGO and consolidative lesions is a predictor of disease worsening [12]. Studies have found that pericardial effusion may occur more frequently in critically ill patients with severe inflammation [29, 30], which is congruent with our findings as pericardial effusion is more prevalent in nonsurvivor ICU cases. In a previous study conducted in Iran, 26.8% of hospitalized patients had cardiomegaly, which is less frequent than what we reported (63.6%) [31]. This can show a higher prevalence of cardiomegaly in ICU-admitted patients than patients admitted to general wards. In another study that compared the radiologic characteristics of critically ill patients with noncritically ill patients, pericardial and pleural effusion were significantly more prevalently seen in patients with severe forms of infection. Furthermore, that study reported that CT scores are higher in critically ill patients, which is not the case in our study [32]. This can be due to the difference in when to consider a patient critically ill and the criteria according to which patients are ICU admitted. Higher CT scores in nonsurvivors also were found in another study that compared survived hospitalized COVID-19 patients with deceased patients (median of 10 vs. 4, *p* < 0.001) [33]. Higher CT scores in all ICU-admitted patients can partly explain this CT score indifference between survivors and nonsurvivors (11.9 vs. 11.4, *p* : 0.59).

In a previously published study, history of heart failure and COPD, clinical findings (SpO_2_ (<92%) and heart rate (>117 bpm)), laboratory findings (procalcitonin (>0.34 ng/ml) and LDH (>460 U/L)), and demographic findings (age (>63 years)) were factors capable of predicting in-hospital mortality [34]. In that study, 641 COVID-19 hospitalized patients were investigated, among which 82 died. In the nonsurvivor group, only 34 patients died after ICU admission, explaining why their results are different from ours. Besides, they did not study radiologic findings. In a prospective cohort study performed in Spain, only two factors, including higher APACHE-II on admission and higher age, were reported as predictors of ICU mortality [35]. In another retrospective cohort study, preexisting hypertension, moderate or severe ARDS, lymphocyte counts of <0.5 × 10^9^/L, albumin of <22 g/L, procalcitonin of >0.2 ng/mL, D-dimer of >1200 ng/mL, and the need for continuous renal replacement therapy were associated with higher mortality in ICU patients [36]. In that study, only 10 out of 103 patients had a CT scan, and just two imaging features were evaluated, including bilateral infiltration and GGO. In a retrospective cohort study of 60 critically ill patients in Wuhan, diabetes, emphysema, higher CRP, neutrophil-to-lymphocyte ratio, and medial or parahilar lung involvement in CT scan were associated with higher death rates [37]. In another study in Wuhan that included 289 hospitalized patients, advanced age, higher CRP levels, the higher number of affected lobes, dyspnea, and smoking were related to higher mortality rate [38]. CT findings reported in their study were GGO, subpleural lesions, and the number of affected pulmonary lobes. Surprisingly, the laboratory findings were not significantly different between survivors and nonsurvivors and were not a predictor of death in ICU-admitted COVID-19 patients according to their study. Laboratory test results change during hospitalization, which can explain the different conclusions drawn by different studies [39]. Moreover, differences between the severity of the disease, studied variables, length of follow-up, inclusion and exclusion criteria, sample size, rate of missing data, laboratory kits, and reservoir time all can partly take effect in this controversial matter [40, 41].

COVID-19 pandemic is challenging healthcare systems around the world. The need for ICU care has been raised dramatically in a short period. In a considerable number of previous studies, the prognostic factors predicting outcome in hospitalized patients (not ICU patients) have been evaluated. To the best of our knowledge, the predictive factors of in-ICU mortality in critically ill patients have not yet been comprehensively studied, including all demographic, clinical, and paraclinical findings to find the confounders and achieve the most reliable model. Most of the studies did not include radiologic findings in their investigation, and if they did, they just considered a few imaging features without demographic and clinical data incorporated. Enrolment of ICU patients, treatment with the same guideline by the same team, and evaluation of images by the same radiologists indicate the homogeneity of our sample as the main strength of this study. Our study had some limitations. First, some habitual factors such as obesity and smoking are believed to be important in the prognostication of COVID-19 patients, and we were not able to assess their impact on the model. Second, the severity of comorbidities and if they are under control or not is more informative than merely reporting their presence. Third, some specific laboratory tests were done in some patients where they were clinically indicated and were not available for all studied patients. Also, we did not have information about treatments that the patients received out of the hospital and the duration between symptoms onset and hospitalization. More studies with a larger number of cases enrolled and more variables included will help to design better prediction models.

## 5. Conclusion

In this study, we designed a model to predict the mortality rate in ICU-admitted COVID-19 patients combining clinical and radiological features including SpO_2_, pericardial effusion, and hypertension. Demographic and laboratory factors did not significantly impact the predictability of the model. This model can help engaged practitioners to pick out high-risk patients for an earlier triage and better resource allocation. Also, it can be used to make more confident decisions on hospitalization, ICU admission, and treatment protocols. Further studies and meta-analyses can help formulating the model in a way that it can be employed in daily practice.

## Figures and Tables

**Table 1:** Details of demographic and clinical data of patients according to their survival status.

Variables	All patients, *N* = 121	Survivors, *N* = 41	Nonsurvivors, *N* = 80	*P* value
Demographic data				
Age^*∗*^	62.2 (14.0)	56.7 (11.7)	65.0 (14.33)	0.002
Gender				
Male	82 (67.8)	29 (70.7)	53 (66.3)	0.62
Female	39 (32.2)	12 (29.3)	27 (33.8)	

Clinical data				
Vital signs^*∗*^				
RR	25.0 (6.8)	26.7 (5.1)	24.3 (7.4)	0.07
SpO_2_	82.7 (9.1)	85.8 (5.3)	81.2 (10.2)	0.01
Systolic BP	124.3 (22.5)	127.2 (21.4)	123.0 (22.9)	0.38
Diastolic BP	73.9 (12.7)	78.2 (10.9)	72.0 (13.0)	0.02
PR	96.4 (16.8)	100.6 (16.2)	94.7 (16.8)	0.10
Temperature	37.6 (0.8)	37.7 (0.9)	37.5 (0.8)	0.46
Hospitalization duration^*∗*^				
Total admission days	15.3 (9.4)	15.8 (6.8)	15.2 (10.0)	0.80
ICU days	9.5 (9.2)	6.0 (2.1)	10.3 (10.0)	0.10
Underlying disease				
HTN	43 (41.3)	8 (25.8)	35 (47.9)	0.03
DM	37 (35.6)	13 (41.9)	24 (32.9)	0.38
COPD	9 (8.7)	5 (16.1)	4 (5.5)	0.08
Immunocompromised	10 (9.6)	3 (9.7)	7 (9.6)	0.99
Hypothyroidism	7 (6.7)	3 (9.7)	4 (5.5)	0.43
Laboratory findings^*∗*^				
WBC	8.9 (4.6)	9.1 (4.6)	8.9 (4.6)	0.81
Neutrophil	7.0 (3.8)	7.1 (4.1)	7.0 (3.6)	0.96
Lymphocyte	1.4 (2.1)	1.3 (0.8)	1.4 (2.5)	0.82
Hemoglobin	12.7 (2.7)	13.1 (2.5)	12.5 (2.8)	0.25
Platelet	212.0 (101.7)	221.2 (108.3)	207.1 (98.5)	0.49
Cr	1.7 (1.5)	1.7 (1.3)	1.7 (1.7)	0.90
Urea	59.2 (59.7)	47.9 (36.9)	65.3 (68.3)	0.15
INR	1.3 (0.7)	1.2 (0.9)	1.4 (0.8)	0.09
PTT	39.6 (17.5)	37.8 (10.9)	40.5 (19.9)	0.48
D-dimer	3579.0 (3409.2)	4086.1 (3746.3)	2767.6 (2995.1)	0.52
LDH	721.0 (356.5)	785.2 (323.0)	688.9 (371.7)	0.32
CRP	127.0 (75.8)	128.3 (77.2)	126.4 (75.6)	0.90
Pro-BNP	6004.3 (10301)	6877.8 (13124.7)	9209.0 (2377.7)	0.79

^*∗*^Reported as mean (standard deviation), all other variables reported as *N* (%). RR = respiratory rate; BP = blood pressure; PR = pulse rate; HTN = hypertension; DM = diabetes; ICU = intensive care unit; WBC = white blood cell; Cr = creatinine; INR = international normalized ratio; PTT = partial thromboplastin time; LDH = lactate dehydrogenase; CR = C-reactive protein; Pro-BNP = pro-B-type natriuretic peptide.

**Table 2:** Radiologic findings stratified based on survival status.

Variables	All patients, *N* = 121	Survivors, *N* = 41	Nonsurvivors, *N* = 80	*P* value
PI scores^*∗*^				
RUL total score	2.3 (1.1)	2.2 (1.0)	2.3 (1.2)	0.82
RML total score	1.8 (1.1)	1.6 (0.8)	1.8 (1.2)	0.43
RLL total score	2.5 (1.2)	2.5 (1.0)	2.6 (1.2)	0.62
LUL total score	2.2 (1.1)	2.2 (1.0)	2.3 (1.1)	0.85
LLL total score	2.5 (1.1)	2.5 (0.9)	2.5 (1.3)	0.84
Total lung GGO score	8.0 (4.3)	7.9 (4.5)	8.0 (4.2)	0.87
Total lung consolidation score	3.6 (3.7)	3.4 (3.5)	3.8 (3.8)	0.61
Total PI score	11.7 (4.8)	11.4 (4.1)	11.9 (5.1)	0.59

PI density index^*∗*^	2.4 (0.8)	2.4 (0.7)	2.4 (0.9)	0.90

Predominant pattern				
GGO	87 (71.9)	29 (70.7)	58 (72.5)	0.84
Consolidation	34 (28.1)	12 (29.3)	22 (27.5)	

Dominant distribution of lesions				
Peripheral	47 (38.8)	14 (34.1)	33 (41.3)	0.59
Axial	34 (28.8)	11 (26.8)	23 (28.7)	
Diffuse	40 (33.1)	16 (39.0)	24 (30.0)	

No. of involved lobes	4.6 (0.8)	4.6 (09)	4.7 (0.8)	0.59

Laterality				
Unilateral	2 (1.7)	1 (2.4)	1 (1.3)	0.62
Bilateral	119 (98.3)	40 (97.6)	79 (98.8)	

Lower lobes involvement				
Yes	114 (94.2)	38 (92.7)	76 (95.0)	0.68
No	7 (5.8)	3 (7.3)	4 (5.0)	

Additional findings				
Cardiomegaly	77 (63.6)	21 (51.2)	56 (70.0)	0.04
Pleural effusion	30 (24.8)	5 (12.2)	25 (31.3)	0.02
Pericardial effusion	13 (10.7)	1 (2.4)	12 (15.0)	0.03
Dilated pulmonary trunk	15 (17.0)	3 (13.6)	12 (18.2)	0.62

Other morphologies				
Parenchymal band	58 (47.9)	22 (53.7)	36 (45.0)	0.37
Crazy paving	54 (44.4)	17 (41.5)	37 (46.3)	0.61
Reverse halo	11 (9.1)	6 (14.6)	5 (6.3)	0.13

^*∗*^ Reported as mean (standard deviation), all other variables reported as *N* (%). PI = pulmonary involvement; RUL = right upper lobe; RML = right middle lobe; RLL = right lower lobe; LUL = left upper lobe; LLL = left lower lobe; GGO = ground-glass opacity.

**Table 3:** Binary backward logistic regression of all clinical findings for predicting death.

Variable	Regression		
	Exp (B)	(95% CI)	*p* value
SpO_2_	0.91	(0.84–0.97)	0.006
Pericardial effusion	6.56	(0.72–59.3)	0.09
Hypertension	4.11	(1.39–12.2)	0.01

CI = confidence interval.

## Data Availability

The data used to support the findings of this study are available from the corresponding author upon request.
